# Antioxidant, Antimicrobial, and Anticancer Effects of *Anacardium* Plants: An Ethnopharmacological Perspective

**DOI:** 10.3389/fendo.2020.00295

**Published:** 2020-06-12

**Authors:** Bahare Salehi, Mine Gültekin-Özgüven, Celale Kirkin, Beraat Özçelik, Maria Flaviana Bezerra Morais-Braga, Joara Nalyda Pereira Carneiro, Camila Fonseca Bezerra, Teresinha Gonçalves da Silva, Henrique Douglas Melo Coutinho, Benabdallah Amina, Lorene Armstrong, Zeliha Selamoglu, Mustafa Sevindik, Zubaida Yousaf, Javad Sharifi-Rad, Ali Mahmoud Muddathir, Hari Prasad Devkota, Miquel Martorell, Arun Kumar Jugran, William C. Cho, Natália Martins

**Affiliations:** ^1^Student Research Committee, School of Medicine, Bam University of Medical Sciences, Bam, Iran; ^2^Department of Food Engineering, Faculty of Chemical and Metallurgical Engineering, Istanbul Technical University, Istanbul, Turkey; ^3^Department of Gastronomy and Culinary Arts, School of Applied Sciences, Özyegin University, Istanbul, Turkey; ^4^Bioactive Research & Innovation Food Manufac. Indust. Trade Ltd., Istanbul, Turkey; ^5^Laboratory of Applied Mycology of Cariri, Department of Biological Sciences, Cariri Regional University, Crato, Brazil; ^6^Laboratory of Planning and Synthesis of Drugs, Department of Antibiotics, Federal University of Pernambuco, Recife, Brazil; ^7^Laboratory of Microbiology and Molecular Biology, Department of Biological Chemistry, Regional University of Cariri, Crato, Brazil; ^8^Department of Agronomy, SAPVESA Laboratory, Nature and Life Sciences Faculty, University Chadli Bendjedid, El-Tarf, Algeria; ^9^State University of Ponta Grossa, Department of Pharmaceutical Sciences, Ponta Grossa, Paraná, Brazil; ^10^Department of Medical Biology, Faculty of Medicine, Nigde Ömer Halisdemir University, Campus, Nigde, Turkey; ^11^Osmaniye Korkut Ata University, Bahçe Vocational School, Department of Food Processing, Osmaniye, Turkey; ^12^Department of Botany, Lahore College for Women University, Lahore, Pakistan; ^13^Phytochemistry Research Center, Shahid Beheshti University of Medical Sciences, Tehran, Iran; ^14^Department of Horticulture, Faculty of Agriculture, University of Khartoum, Shambat, Sudan; ^15^School of Pharmacy, Kumamoto University, Kumamoto, Japan; ^16^Program for Leading Graduate Schools, Health Life Science: Interdisciplinary and Glocal Oriented (HIGO) Program, Kumamoto University, Kumamoto, Japan; ^17^Department of Nutrition and Dietetics, Faculty of Pharmacy, Centre for Healthy Living, University of Concepción, Concepción, Chile; ^18^Unidad de Desarrollo Tecnológico, Universidad de Concepción UDT, Concepción, Chile; ^19^G. B. Pant National Institute of Himalayan Environment and Sustainable Development, Garhwal Regional Centre, Uttarakhand, India; ^20^Department of Clinical Oncology, Queen Elizabeth Hospital, Hong Kong, China; ^21^Faculty of Medicine, University of Porto, Alameda Prof. Hernâni Monteiro, Porto, Portugal; ^22^Institute for Research and Innovation in Health (i3S), University of Porto, Porto, Portugal

**Keywords:** *Anacardium*, cashew nut, phytotherapy, antioxidant, antimicrobial, anticancer

## Abstract

*Anacardium* plants have received increasing recognition due to its nutritional and biological properties. A number of secondary metabolites are present in its leaves, fruits, and other parts of the plant. Among the diverse *Anacardium* plants' bioactive effects, their antioxidant, antimicrobial, and anticancer activities comprise those that have gained more attention. Thus, the present article aims to review the Anacardium plants' biological effects. A special emphasis is also given to their pharmacological and clinical efficacy, which may trigger further studies on their therapeutic properties with clinical trials.

## Introduction

*Anacardium* plants have received an increasing attention in recent years. Among the *Anacardium* plants*, Anacardium occidentale* (cashew apple) leaf extract is traditionally used in treating various diseases in tropical America, especially in North-Eastern Brazil. Indeed, the popular drinks in Brazil include fresh and processed cashew apple juice. Cashew plants have been used for centuries as folk medicine in South America and West Africa. Quite a number of biological properties have been reported, among them antimicrobial, antioxidant, antiulcerogenic, and anti-inflammatory effects have drawn public attention. In Nigeria, these species have also been used to treat cardiovascular disorders. While in Brazil, these species are used as infusion for curing ailments (1).

*Anacardium* species contain various secondary metabolites in its leaf and shoot powder, fruits and other parts of the plant, which can be used regarding their nutraceutical, medicinal and biological aspects (Table 1).

**Table 1:** Bioactive contents of *Anacardium* plants.

**Compounds**	***Anacardium* plant**	**Part of the plant**	**Amount**	**Reference**
Phenols	*A. occidentale*	Leaf	847.41 mg GAE^a^/100 g fw^b^	(2)
	*A. othonianum*	Edible parts	160.74 mg GAE/100 g fw	(3)
Anthocyanin	*A. occidentale*	Leaf	0.37 mg/100 g fw	(2)
		Pulp	9.5 mg/100 g fw	(4)
Carotenoids	*A. occidentale*	Leaf	5.42 βCE/100 g fw	(2)
		Pulp	0.4 mg/100 g fw	(4)
Ascorbic acid	*A. occidentale*	Leaf	494.43 mg/100 g fw	(2)
		Pulp	190 mg/100 g fw	(4)
	*A. othonianum*	Edible parts	5.48 mg/100 g fw	(3)

a *GAE, Gallic acid equivalent*;

b *fw, fresh weight*.

Interestingly, cashew fruit is tasty and rich in minerals, vitamins, and some essential nutrients. It has high vitamin C, nearly to five times higher than oranges and also with high minerals content. The fruit comprises of some volatile compounds, e.g., esters, terpenes, and carboxylic acids (5). Cashew bark and leaves have a rich amount of tannins (6). Cashew nut kernel testa contains tannin as an interesting economical source of antioxidants that can be used for both food and nutraceutical purposes (7). The species also contain a rich amount of flavonoids with diverse physiological effects. Anacardic acids were detected in higher amount in nutshells. Cardanol (decarboxylated anacardic acid) and cardol are found as the main components of commercial cashew nut shell liquid. Cardanol is extensively used as a synthon for the synthesis of several polymers and agricultural products (8). Cashew nut shell liquid extracted by solvent is a mixture of alkenyl phenols, including anacardic acid. As defatted, cashew kernel flour is a good source of protein and minerals. Furthermore, it can serve as low-fat fabricated food and animal feed. Animal or poultry feeds are mostly formulated using a substantial amount of cashew fiber. Besides, cashew fiber along with cashew nut shell liquid, both possess high anacardic acids contents and therefore can be utilized in functional food formulations (9).

## *Anacardium* Plants. Key Focus on Biological Effects

Herbal treatments are the most popular form of traditional medicine and commonly used as primary health care (10). All parts of cashew tree (mainly leaf and stem bark) have been extensively used as traditional herbal medicine, contributing health benefits all over the world (Table 2) (12, 37, 38). Thus, in the last decades, *Anacardium* plants folk medicinal properties, and multiple biological effects being studied extensively (Tables 3, 4).

**Table 2:** Traditional medicinal uses of different *Anacardium* plant parts.

**Part of the plant used**	**Medicinal uses, preparation, and applications**	**Locations**	**Reference**
Bark	Hemorrhoids and severe diarrhea	Brazil	(11)
	Treatment of lower extremity pains and skin injury; exerts anti-inflammatory effects	Pataxó Indians, Xucuru Indians, Africa	(12–14)
	Treatment of type 2 diabetes	Portugal	(15)
	Strengthened the womb by washing of the decoction	Guatemala	(16)
	Treatment of inflammation of extremities, usually as hot baths	Panama	(17)
	Enteric condition, worms	Nigeria	(18)
	Rheumatic diseases	Brazil	(6)
	Diarrhea, fever, skin rashes, and sore. Topical for aches and pains	Nicaragua	(19, 20)
	Used for infectious, inflammatory and oxidative stress conditions	Nigeria	(21, 22)
	To relieve toothache and sore gums, treat dysentery, diarrhea and piles, as also to treat pellagra	Tropical Africa, Ghana	(23, 24)
	Allergy, yellow fever, eye pains, external and internal wounds, stomach ach (dysentery, diarrhea of children), cough, teeth pains (teeth bleeding, caries), hypertension, diabetes, hemorrhoid, sexual weakness	Benin	(25)
	Anti-ulcerous	Africa	(12)
Leaves	Diarrhea, fever, skin rashes, and sore. Topical for aches and pains	Nicaragua	(19, 20)
	Enteric condition, worms	Nigeria	(18)
	Cancerous diseases	Nigeria	(26)
	Dysentery, pain-killers, venereal diseases	Africa	(27)
	Anti-hypertensive	Indonesia	(28)
	Malaria and yellow fever as well as diarrhea	Malaysia	(29, 30)
	Blisters, itching, ulcers, and warts	India	(31)
	Rheumatic disorders and hypertension	Indonesia. Malaysia	(2, 28)
	Gastrointestinal disorders (acute gastritis, diarrhea), mouth ulcers, and throat problems	West Africa and South America	(18, 29, 30)
	Eczema, genital problems, venereal diseases, impotence, bronchitis, cough, and syphilis-related skin disorders	Brazil	(32)
	Toothache and sore gums, dysentery, diarrhea, and piles, and to treat pellagra	Tropical Africa, Ghana	(23, 24)
	Fever, malaria, dysentery, teeth's caries, cough, and hypertension	Benin	(25)
	Dysentery, diarrhea, piles, toothache and sore gums. Uses for remedy of rheumatism and hypertension	Southeast Asia	(2, 28, 29)
Root	Cough, stomach pain, tooth decay, hypertension, and malaria	Benin	(25)
	Diarrhea, stomach pains, and as purgative	Guatemala	(12, 16)
Buds	Asthma.	Guatemala	(16)
Stalk	Teeth's caries as toothpick	Benin	(25)
Apple	Scorpion and bee sting, application of juice at the sting	Benin	(25)
Cashew apple juice	Syphilis, cholera and kidney disease, as antiscorbutic, astringent and diuretic	Africa	(12)
Liquid nuts	Tinea as ointment	Benin	(25)
	Mental derangement, heart palpitation and rheumatism	Africa	(12)
Kernel	Demulcent and emollient and for diarrhea	Africa	(12)
Nut oil	Antifungal and for healing cracked heels, antihypertensive and purgative; for blood sugar, kidney diseases, cholera, hookworms, corns, and warts	Benin, Brazil, Africa	(12, 33)
Cashew gum	Anti-inflammatory, analgesic, antiasthmatic, and antidiabetic agent; for gastrointestinal diseases, including diarrhea, warts, coughs, and wounds.	Brazil	(34–36)
Cashew syrup	Coughs and colds	Africa	(12)

**Table 3:** Pharmacological effects of *Anacardium* species.

***Anacardium* plant**	**Pharmacological effect**	**Reference**
*A. occidentale*	Antioxidant	(9, 39–71)
	Anti-inflammatory	(44, 61, 72–77)
	Antimicrobial	(29, 40, 49, 54, 56, 58, 60, 63, 78–97)
	Antibacterial	(39, 46, 71, 75, 98–110)
	Cytotoxic	(71, 80)
	Diabetic induction	(55, 110–112)
	Hypolipidemic	(50, 64, 67, 113)
	Antimutagenic	(45)
	Analgesic	(70, 72, 73)
	Antityrosinase	(39)
	Genotoxic	(114)
	Hypoglicemic	(64, 102, 115–119)
	Wound healing	(58, 61)
	Acetylcholinesterase activity	(47)
	Anticancer	(97)
	Antiadherent	(78)
	Reduction of dental plaque and gengivitis	(120)
	Insecticidal	(81)
	Antifungal	(121)
	Antisickling	(110)
	Antibiofilm	(79, 90)
	Vermicidal effect in human ancylostomiasis	(122)
	Antivectorial	(123)
	Schistosomicidal	(124)
	Larvicidal	(125–127)
	Ovicidal	(127)
	Renal protective	(117)
	Antihypertensive	(128)
	Antidepressant	(129)
	Anticonvulsant	(130)
	Anthelmintic	(131)
*A. humile*	Antioxidant	(132)
	Antifungal	(133)
*A. microcarpum*	Antioxidant	(1)
	Cytotoxic	(1)
	Antibacterial	(134)
*A. excelsum*	Antimicrobial	(135)
*A. giganteum*	Cytotoxic effect	(136)
*A. othonianum*	Antifungal	(137)
	Cytotoxic	(137)

**Table 4:** *Anacardium* plants tested for bioactives effects and its corresponding extracts.

***Anacardium* plants**	**Extraction**	**Reference**
*A. occidentale*	Ethanol	(43, 44, 49, 53, 54, 63, 68, 71, 75, 77, 80, 83, 85, 86, 88, 89, 92, 93, 97, 98, 100–103, 105, 107–109, 114, 118, 124, 126, 127, 129–131, 138)
	Aqueous	(43, 49–51, 54, 61, 62, 64, 66, 73, 89, 92, 100, 101, 104, 105, 109, 112, 113, 121)
	Petroleum ether	(43, 72, 85, 110)
	n-Hexane	(42, 47, 48, 53, 54, 56, 59, 73, 82, 102, 107, 117, 127, 128)
	Dichloromethane	(56, 73, 92)
	Ethyl acetate	(42, 47, 48, 53, 56, 76, 92, 102, 110)
	n-Butanol	(56)
	Methanol	(29, 39, 40, 42, 48, 50, 55, 60, 67, 70, 72–74, 84, 91, 96, 110, 115, 116, 119, 125)
	Essential oil	(31, 65, 69, 100)
	Acetone	(49, 72, 87, 88)
	Hydroalcoholic extract	(58, 90, 111)
	Chloroform	(50, 72)
*A. humile*	Acetone	(132)
	Methanol	(132)
	Water	(132)
	Hydroalcoholic extract	(132, 133, 139)
*A. microcarpum*	Ethanol	(1, 134)
	Ethyl acetate	(134)
	Methanol	(134)
*A. excelsum*	Ethanol	(135)
*A. giganteum*	Methanol	(136)
*A. othonianum*	n-Butanol	(137)
	Ethanol	(137)
	n-Hexane	(137)

### Antioxidant Activity

Oxidation process produces free radicals which contain unpaired electron. They can cause DNA damages and attack lipids and proteins. Antioxidants can protect free radical-induced damages by transferring electrons or hydrogen. Thus, foods with antioxidants may provide defense against free radical damage in the body and may prolong the shelf life of food products.

Fermented fruit juice of *A. occidentale* was reported with high antioxidant activity (140). Tan and Chan (39) reported that fresh *A. occidentale* leaves exhibit high antioxidant and phenolic contents as assessed by 2,2-diphenyl-1-picrylhydrazyl (DPPH) radical scavenging, potassium ferricyanide, ferric reducing antioxidant power (FRAP), ferrous ion chelating ability, ferrozine, Folin–Ciocalteu, aluminiumchloride, and molybdate assays. Cashew apple juice and pulp have been reported to have considerable amount of vitamin C (141–144). High contents of polyphenols, tannins and dietary fiber were also reported (145). Furthermore, it was reported that copper, iron, zinc, and antioxidant compounds are also present in cashew apple juice, which were more abundant compared to cashew apple fiber (146).

#### *In vitro* Studies

*A. occidentale* revealed high antioxidant activity through DPPH radical scavenging, ferric thiocyanate, and thiobarbituric acid assays. However, it did not exhibit nitric oxide (NO) inhibitory activity (40, 147). Good antioxidant capacity of red and yellow cashew was also observed using 2,2'-azino-bis(3-ethylbenzothiazoline-6-sulfonic acid) (ABTS) and DPPH radical scavenging assays (148). Moreover, Kongkachuichai et al. (149) reported that young cashew leaves demonstrate high antioxidant capacity by oxygen radical absorbance capacity (ORAC) and FRAP assays.

Different parts of *A. occidentale* have strong antioxidant potency. For instance, ethanol extract of cashew nut skin demonstrated high total phenolic content and good antioxidant capacity as assessed by ABTS radical scavenging, superoxide scavenging, deoxyribose oxidation, and lipid peroxidation assays (150, 151). Andrade et al. (152) found that technical cashew nut shell liquid has high antioxidant capacity, perhaps due to its high content of cardanol and cardol, and thus it can be used as a natural antioxidant for nutraceutical and pharmaceutical purposes. The significant correlation between antioxidant capacity and the contents of anacardic acids, cardols, and cardanols was also reported, where anacardic acid content was found higher in cashew apple and fiber, whereas cardols and cardonols contents were higher in cashew nut shell liquid (9).

Antioxidant capacity of *Semecarpus anacardium* is also worth noting. Barman et al. (153) reported that *S. anacardium* nut ethanol extract had high antioxidant activity as assessed by DPPH and ABTS radical scavenging and metal chelating assays. It should be noted that antioxidant activities of plants can be affected by manufacturing process (154). Tan and Chan (39) reported that up to 30% decrease can be obtained in phenolic content and antioxidant activity of *A. occidentale* after blanching. However, no changes due to microwave treatment was observed. Interestingly, Trox et al. (155) reported that bioactive content of cashew nut kernels decreased after conventional shelling techniques, such as oil-bath roasting, steam roasting, drying, and open pan roasting; and they recommended flores hand-cracking method to minimize losses. Moreover, the contents of vitamin C, flavonoids, and polyphenols in cashew apple juice were increased after cold plasma treatment. Yet decreases in bioactive contents were observed at excessive exposure (156). Liao et al. (157) reported that antioxidant activity of cashew nut kernels were not affected by hot air-assisted radio frequency roasting. Moreover, sonication treatment improved the bioactive compound extraction yield from cashew apple bagasse compared to conventional heat treatment, and the optimum conditions were recommended as treatment for 6 min at an intensity of 226 W/cm^2^ and 1:4 bagasse-to-water ratio resulting the highest vitamin C and total phenolic contents (158). In addition, total phenolic and tannin contents of *A. occidentale* were increased by gamma-irradiation (159).

There are changes in the antioxidant and phenolic contents of cashew during ripening. Gordon et al. (41) observed a decrease in phenolic content of cashew apple during ripening compared unripe apple, but ascorbic acid concentration and antioxidant activity was increased during ripening. Thus, it is plausible that the antioxidant activity of cashew apple may depend on ascorbic acid rather than phenolic content (41).

The antioxidant capacity of *anacardium* also depends on the extraction method. Razali et al. (42) reported that *A. occidentale* shoots methanol extract exhibited higher antioxidant activity compared to ethyl acetate and hexane extracts as assessed by ABTS, DPPH, superoxide anion, and NO radical scavenging assays; moreover, total phenolic content of methanol extract was found to be higher. De Abreu et al. (160) reported that carotenoids content of cashew apple was higher compared to aqueous extracts. In another study, it was observed that ethyl acetate extract of *S. anacardium* stem bark exhibited higher phenolic content compared to that of hexane and chloroform extracts (161). Chotphruethipong et al. (162) recommended extraction at 34.7°C for 64 min with ethanol-to-solid ratio of 18:1 (v/w) as optimum conditions for cashew leaves extraction. Aqueous, ethanol and petroleum ether (60–80°C) extract from *A. occidentale* leaves were studied for antioxidant activities through NO production and DPPH radical assays. Ethanol extract revealed the higher potential, followed by aqueous and petroleum ether extracts (43).

The antioxidant effect of *A. occidentale* leaf extract was measured and exerted noticeable activity in treating RAW 264.7 macrophage cells. Leaf extract administration (0.5 and 5 μg/mL doses) reduced oxidative damage in macrophage cells. Moreover, oxidative damage attributes induced in lipopolysaccharide (LPS)-stimulated RAW 264.7 macrophage cells was inverted by leaf extract (44). Antioxidant activity of whole cashew nuts products, treated with low- and high-temperature was also determined. Results indicated that antioxidant activities of cashew nut, kernel, and testa phenolics extracted increased with the increasing roasting temperature. The highest activity was observed in nuts roasted at 130°C for 33 min, as revealed by DPPH, ORAC, Trolox equivalent antioxidant capacity (TEAC), FRAP, and hydroxyl radical scavenging assays (163). Frozen cashew pulps from *A. occidentale* were investigated for antioxidant activity using FRAP and DPPH assays (164).

Methanol, hexane and ethyl acetate extracts of *A. occidentale* shoots were investigated through using ABTS and DPPH radicals, superoxide anion radicals, NO radicals, and ferric ions reducing assays. *A. occidentale* methanol extract was the most potent reducing agent and radical-scavengers. In case of ethyl acetate extract, some antioxidant effects were detected, and hexane extract was the least reactive. Methanol extract revealed 7-fold higher total phenolic content than the hexane and ethyl acetate extracts, suggesting the possible contribution of phenolics in the observed effects (42). *Anacardium microcarpum* antioxidant effects were also investigated on human leukocytes and erythrocytes using *in vitro* methods. The half maximal inhibitory concentration (IC_50_), for DPPH, varied from 27.9 (ethyl acetate fraction) to 32.9 μg/mL (ethanol fraction), and Fe^2+^ (10 μM)-induced lipid peroxidation was strongly inhibited by all fractions in rat brain and liver homogenates. Interesting, all the studied fractions were not cytotoxic to leukocytes and were able to inhibit against H_2_O_2_-induced cytotoxicity. No effects was found on human erythrocytes osmotic fragility, thus suggesting that *A. microcarpum* infusion can be safely consumed (1).

#### *In vivo* Studies

Technical cashew nut shell liquid decreased oxidative stress induced by paraquat or H_2_O_2_ exposure in *Saccharomyces cerevisiae*, demonstrating the antioxidant activity in the *in vivo* model assessed by DPPH scavenging and xanthine oxidase assays (152). Moreover, Encarnação et al. (15) observed antioxidant activity of *A. occidentale* stem bark by total phenolic content and DPPH scavenging assays, and they also reported that an oral dose of 2,000 mg/kg does not possess genotoxicity as assessed by *in vivo* tests on mice. Pereira et al. (165) observed antioxidant activity in rats fed with mixed tropical fruit juices containing 5% cashew apple.

The antioxidant capacity of *Anacardium* plants can also be related to the antimicrobial and anticancer properties. For instance, Premalatha and Sachdanandam (166) reported that *S. anacardium* nut extract has anticancer properties, and it was related to the high antioxidant capacity of the product, as it triggered antioxidant defense system in an *in vivo* study performed on male Wistar rats. The antioxidant effects of *S. anacardium* were also investigated by Ramprasath et al. (167) on arthritic rats. The results of this study shows that *S. anacardium* extract restored the increment in C-reactive protein and erythrocyte sedimentation rate observed in arthritic animals.

### Antimicrobial Activity

The ethnomedicinal use of *Anacardium* plants for the treatment of bacterial and fungal infections is practically limited to *A. occidentale* (Table 5). The applicability of this plant for these therapeutic purposes has been reported in South America, Central America, Africa, and Asia. The bark is commonly used, although the leaves, roots, seeds, and fruits also can be utilized. Despite many ethnobiological studies omitting information regarding preparation, decoction is more incidental and oral administration predominates. The species is mainly used for gastrointestinal and skin disorders, where these can be caused by action of various bacterial and fungal species. On the other side, the adaptive capacity of microorganisms has contributed to their resistance to current drugs available, with this encouraging the search for new antimicrobial substances (179).

**Table 5:** Traditional medicinal uses of *Anacardium* plants related to bacterial and fungal infection.

**Species**	**Applications**	**Part of teh plant**	**Preparations**	**Method**	**Location**	**Reference**
*A. occidentale*	Dysentery and stomach ache	Bark	Infusion and soak in water	Oral use	Brazil	(168)
	Aphtha	NI	NI	NI	Brazil	(169)
	Infectious processes	Bark	Maceration	Oral use	Brazil	(170)
	Rheumatism	Bark, leaves	Sauce, decoction	NI	Brazil	(171)
	Skin infection, dysentery, diarrhea, thrush	Leaves, root, bark	Infusion	NI	Nigeia	(172)
	Stomach ulcer	Bark	Decoction	Oral	Cuba	(37)
	Stomach ulcer, wounds.	Leaves	NI	NI	Madagascar	(173)
	Asthma	Fruit	Raw	Oral	India	(174)
	Wound	Root, seeds, fruit	NI	NI	India	(175)
	Oral syphilis	Unripe fruits	Decoction	Mouth rinse	Cameron	(176)
	Stomach ache	Bark	Maceration	Oral	Nigeria	(177)
*Anacardium* sp	Diarrhea, skin wounds	NI	NI	NI	Brazil	(178)

#### *In vitro* Studies

##### Antibacterial properties

In antibacterial property of medicinal plants from Nigeria, *A. occidentale* hydroethanolic extracts (leaf/bark) showed positive effects against *Escherichia coli, Staphylococcus aureus, Enterobacter* species, *Streptococcus pneumoniae, Corynebacterium pyogenes, Enterococcus faecalis*, multiresistant *S. aureus, Acinetobacter* species, *Pseudomonas aeruginosa*, and multiresistant *P. aeruginosa* during cavity diffusion tests with inhibition halos varying from 6 to 14 mm (18).

In the study by Akinpelu (29), *A. occidentale* bark methanol extract (60%) exhibited antimicrobial activity against 13 out of 15 bacterial isolates, obtaining the activity against *Shigella dysenteriae* and *Klebsiella pneumoniae*, using the agar and broth microdilution methods (20 mg/mL). In the study by Melo-Cavalcante et al. (45), the antibacterial effect of fresh (25, 50, and 100 μL/plate) and processed (100, 500, and 2,000 μL/plate) cashew juices (*A. occidentale*) were assessed against *Salmonella typhimurium*, and all tested doses revealed to be effective. Melo et al. (180) demonstrated the action of the *A. occidentale* stem bark hydroalcoholic extract against *Streptococcus mitis, Streptococcus mutans*, and *Streptococcus sanguis* using diffusion and microdilution techniques. The extract concentrations (50 to 0.04 mg/mL) presented halos varying from 19 to 0 mm for *S. mitis*, 16 to 20 mm for *S. mutans* and 18 to 20 mm for *S. sanguis*. Chlorhexidine (0.12 to 0.001875%) obtained inhibition halos of 14 to 12 mm. The bacterial surface adherence analysis revealed the extract interferes with adhesion at 0.31 and 0.15 mg/mL.

Silva et al. (181) demonstrated that methicillin resistant and sensitive *S. aureus* samples were sensitive to pure (100 mg/mL) and diluted (1:2-1:64) *A. occidentale* stem bark extract, presenting inhibition halos ranging from 10 to 20 mm. The norfloxacin control inhibition halos ranged from 11 to 36 mm.

The liquid from *A. occidentale* cashew bark was evaluated as a food additive for ruminants, where prior to its addition to feed, cashew nut shell liquid was tested by microdilution (0 to 50 μg/mL). The lower MIC values were obtained (1.56–6.25 μg/mL) against *Ruminococcus flavefaciens, Ruminococcus albus, Ehrlichia ruminantium*, and *Butyrivibrio fibrisolvens* and moderate values (25 to 50 μg/mL) against *Streptococcus bovis* and *Lactobacillus ruminis*. Four of the tested bacteria (*Succinivibrio dextrinosolvens, Ruminobacter amylophilus, Elenomonas ruminantium*, and *Megasphaera elsdenii*) were insensitive to cashew nut shell liquid (MIC ≥ 50 μg/mL). The bacteria *Fibrobacter succinogenes, Prevotella ruminicola*, and *Succinimonas amylolytica* were sensitive to the cashew nut shell liquid (MIC: 3.13 to 12.5 μg/mL). Thus, cashew nut shell liquid inhibits rumen-specific bacteria and its activity is promising (182).

The dried extract obtained from *A. occidentale* leaf powder dye (20%; 200 mg/mL) showed an effect against *S. aureus* which produced the largest inhibition halo (12 mm). In comparison, gentamicin and chloramphenicol produced halos of 20 and 21 mm, respectively (98).

Campos et al. (183) evaluated two *A. occidentale* starch (10 to 60 mg/mL) samples (crude and purified) against *E. coli, S. aureus, Listeria innocua, P. aeruginosa, Enterococcus faecium*, and *Lactobacillus acidophilus* strains. Both samples were able to inhibit growth, with MICs ranging from 20 to 30 mg/mL for the crude starch, and 40 to 60 mg/mL for the purified starch. The result was obtained for *P. aeruginosa* (20 mg/mL). In the subculture assay (Minimum bactericidal concentration, MBC), only the purified starch sample displayed action at 50 mg/mL. In the cell's structural analysis, changes such as pili loss and cell lysis (10 mg/mL) were observed.

Kaewpiboon et al. (184) confirmed the action of *A. occidentale* dry leaf ethanolic extract (5%) by disc-diffusion and microdilution, obtaining 15- and 13-mm inhibition halos and MICs of 250 and 500 μg/mL against *E. coli* and *P. aeruginosa*, respectively. Chloramphenicol (20 μg/disc) obtained a diameter varying from 15 to 30 mm and a MIC ranging from 7.1 to 125 μg/mL.

The methanolic and n-hexane extracts from *A. occidentale* aerial parts showed inhibitory effects against bacteria (*S. aureus, E. faecalis, E. coli, P. aeruginosa, K. pneumoniae*, and *Mycobacterium smegmatis*) with MICs ranging from 62.5 to 250 μg/mL (n-hexane) and 7.5 to >250 μg/mL (MeOH), with the effect for both extracts being obtained against *S. aureus* (185). *A. occidentale* bark ethanolic extract (3.125, 6.25, 9.375, and 12.5 mg/mL) was investigated against *Streptococcus sanguinis* biofilm formation. The extract inhibited biofilm formation as the concentration increased, ranging from 67.22 to 94.20%. The chlorhexidine control (0.12%) presented 89.55% inhibition (186).

An *A. occidentale* tincture (20%) obtained from a homeopathic Pharmacy was tested for oral bacteria biofilm-forming inhibition, and MICs of 3.12 and 0.78 mg/mL were obtained against *S. mutans* and *Streptococcus oralis* by microdilution (187). As for the diffusion, the tincture (pure 1:1) from the same *Anacardium* species obtained from a Manipulation Pharmacy (diluted in 20% in 70% alcohol) showed inhibition halos of 12, 13, 11, and 15 mm against *S. mutans, S. salivary, E. faecalis*, and *Eikenella corrodens*, respectively. For Chlorhexidine, the values obtained were 17, 15, 17, and 19 mm respectively (188).

Menezes et al. (78) extracted tannins from *A. occidentale* stem bark and evaluated by cavity diffusion its antibacterial effect against *S. mutans, S. mitis, S. sanguis, Streptococcus salivarius*, and *Lactobacillus casei* at 1:1, 1:2, to 1:16 μg/mL, with inhibition halos ranging from 11 to 17 mm. In the presence of 5% sucrose, the bacterial anti-adherence effect was observed using concentrations from 1:8 to 1:512 μg/mL, obtaining in some cases, a better effect than 0.12% chlorhexidine gluconate (1:16–1:32 μg/mL).

Fresh and processed (bleached and irradiated) leaves extracts of *A. occidentale* showed antibacterial effect. The minimum inhibitory doses capable of forming inhibition halos were 0.06 to 0.50 mg/disk, having an effect against *Brevibacillus brevis, Micrococcus luteus, Staphylococcus cohnii, E. coli*, and *P. aeruginosa* with the best performance being obtained for the irradiated extract (39). The *A. occidentale* leaf extract was investigated by Ayu et al. (189) against *Aggregatibacter actinomycetemcomitans*, a bacterium responsible for gingivitis. Inhibition zones ranging from 4.47 to 8.05 mm were observed for the tested concentrations (8, 41, 145, 164, 189, 190), and 96%) using agar diffusion method. The metronidazole control displayed a halo of 13.91 mm.

The cashew pulp juice extract (1 at 7.8 mg/mL) was tested against *S. aureus* planktonic cells and for the first time, against *S. aureus* biofilms where the cashew pulp juice extract was also tested in association with antimicrobials using the broth microdilution method and MBC. A MIC of 15.6 μg/mL, a MBC of 125 μg/mL and a biofilm Eradication Concentration of 500 μg/mL were obtained, demonstrating its antimicrobial and antibiofilm activity (79). The crude *A. occidentale* hydroalcoholic extract bark also showed good effects against *S. aureus* through disk diffusion (20 μL), presenting an inhibition halo of 11 mm (191).

In the study by Muraina et al. (192), *A. occidentale* leaf extract (10,000 μg/mL) was used against *Mycoplasma* spp., using the broth microdilution method. The antibiotic tylosin (1,280 μg/mL) was used as a positive control and acetone as a negative control. The authors obtained a significant result for the extract as an anti-mycoplasm product (MIC = 310 μg/mL).

Cajado et al. (193) investigated the aqueous and hydroalcoholic *A. occidentale* dry leaf limb extract against *E. coli, S. aureus*, and *K. pneumoniae* strains. The agar diffusion technique demonstrated *S. aureus* inhibition at 75 and 150 mg/mL (aqueous: 9.5 mm and hydroalcoholic: 8 and 10 mm). Moreover, amoxicillin in association with clavulanic acid (30 μg/mL) presented an action ranging from 12 to 38 mm.

Harsini (194) investigated the action of *A. occidentale* stem bark ethanolic extract against *S. aureus* by analyzing Ca^2+^ and K^+^ ion leakage inside the bacterial cell. Ca^2+^ leakage at 0% (control), 3, 5 and 7% concentrations varied from 2.42 to 66.73 mM, and for K^+^ this ranged from 15.28 to 1,251 mM, destabilizing the cell.

Quelemes et al. (195) evaluated the *A. occidentale* cashew starch (CG) antibacterial effect by microdilution, as well as that of its quaternized derivatives (QCG-1, QCG-2, and QCG-3) against a series of bacteria. QCG-2 and QCG-3 presented antimicrobial activity against *S. aureus* and *Staphylococcus epidermidis* (standard and resistant) where a MIC of 31.5 to 250 μg/mL and a MBC of 62.5 to 500 μg/mL were obtained. These results show the quaternized derivatives may be a promising tool in the development of biomaterials with antiseptic action.

The purified *A. occidentale* bark liquid was able to inhibit *Bacillus subtilis* growth (0.6%) and alter its morphology (0.4%). The activity of the purified cashew nut shell liquid was tested using the colony counting method, where an IC_50_ of 0.35% (v/v) was observed, presenting a bactericidal effect as well as cellular elongation suggesting bacterial cell division proteins may be a cashew nut shell liquid target (196).

Dos Santos et al. (197) obtained crude and fractionated extracts [hexane, dichloromethane, ethyl acetate, and methanol: ethyl acetate (9:1)] from *A. occidentale* leaves and evaluated these before and after being irradiated with gamma radiation, showing its effect over several *S. aureus* species was intensified after gamma radiation exposure (non-irradiated: MIC of 500 to >2,000 μg/mL, irradiated: MIC of 250 to >2,000 μg/mL).

De Araujo et al. (198) tested by microdilution, extracts rich in tannins obtained from *A. occidentale* stem bark which inhibited cariogenic bacteria growth from the *Streptococcus* genus, obtaining a MIC of 3,125 μg/mL (*S. mitis, S. mutans*) and of 6.25 μg/mL (*S. oralis, S. salivarius, S. sanguinis*, and *Streptococcus sobrinus*). The 0.12% chlorhexidine control presented MICs ranging from 0.390 to 3,125 μg/mL.

*A. occidentale* cashew bark oil (heated and raw-−1,600 to 0.7812 μg/mL) as well as 16 isolated compounds (anacardic acids, cardols and cardanols) were investigated by Himejima and Kubo (99). Using microdilution method, the following strains were tested: *B. subtilis, Brevibacterium ammoniagenes, S. aureus, S. mutans, E. aerogenes, E. coli, P. aeruginosa*, and *Propionibacterium acnes*. The oil's best result was obtained against *B. subtilis* (heated: 6.25 μg/mL and crude: 12.5 μg/mL) and *S. mutans* (heated: 3.13 μg/mL and crude: 3.13 μg/mL). The isolates obtained MIC values ranging from 0.39 to 100 μg/mL, with *P. acnes* being the most susceptible strain. Kubo et al. (199) isolated from *A. occidentalis* cashew, and tested through microdilution, a series of anacardic acids and (*Z*)-2-alkenyls against *H. pylori*, obtaining MIC values ranging from 200 to 800 μg/mL.

In the study by Green et al. (200), a series of anacardic acid analogs (200 μg/mL) extracted from *A. occidentale* with different side chains were evaluated, where phenolic, branched and alicyclic analogs were synthesized and their antibacterial activity was tested against methicillin-resistant *S. aureus* (MRSA) using microdilution method. The result was obtained for the side chain branched analog, 6-(40,80-dimethylnonyl) salicylic acid, and the side chain alicyclic analog, 6-cyclododecylmethyl salicylic acid (MIC = 0.39 μg/mL), respectively. This activity was greater than that of the most potent isolated antibacterial anacardic acid.

Based on the previous antibacterial anacardic acid study, 6-pentadecenyl salicylic acids isolated from *A. occidentale* cashew tree, a series of 6-alk(en)yl salicylic acids (200 μg/mL) were synthesized and tested for their antibacterial activity against *S. mutans* using broth microdilution. Among these, 6-(40,80-dimethylnonyl) salicylic acid was found to exhibit the most potent antibacterial activity against this cariogenic bacterium with a MIC of 0.78 μg/mL (201).

*S. aureus* and *Streptococcus pyogenes* were sensitive to *A. occidentale* cashew hexane and anacardic acid (both 20 mg/mL) extracts. When using agar diffusion, 18- and 16-mm halos were obtained for the extract and 16 mm for the acid, while microdilution analysis revealed MIC values ranged from 20 to 1:256 mg/mL. The amoxicillin control (20 mg/mL) inhibited total growth in the strains (179).

Anacardic acid (2, 10, 50, and 250 μg/mL) extracted from the cashew bark oil (*A. occidentale*) inhibited *S. aureus* biofilm formation at 40, 76, 80, and 99.96% as the concentration increased. The acid also reduced *S. aureus* adherence to catheters by 20% at the lowest tested dose (202). *A. occidentale* stem bark methanolic extract and isolated compounds (Pinostrobin, Pinocembrin, and 4-hydroxybenzaldehyde) presented inhibition zones, through disc diffusion, varying from 6.43 to 12.56 mm against *Salmonella dysenteriae, Salmonella typhi, S. aureus*, and *E. coli*, with the best results being obtained using Pinocembrin. Chloramphenicol exhibited inhibition zones of 18.71 to 21.50 mm. The IC_50_ varied from 62.5 to 500 (4,098 μM), with the best effect being obtained using the extract against all strains (46).

These studies using the *A. occidentalis* species prioritized the evaluation of hydroethanolic and methanolic extracts, using mainly the stem bark from the species. The method chosen for most of the tests was microdilution, followed by disk diffusion. Moreover, isolated compounds have already been evaluated, mostly anacardic acids. In addition to *A. occidentale*, two other species, *A. microcarpum* and *Anacardium humile* were tested against bacteria.

The crude ethanolic extract, ethyl acetate fraction and methanolic fraction from fresh *A. microcarpum* bark had their intrinsic antibacterial activity evaluated displaying antibacterial activity at 512 μg/mL (*E. coli, P. aeruginosa*, and *S. aureus*), which when combined with antibiotics, potentiated the effect of amikacin and gentamicin against the strains (134).

In another study by Barbosa-Filho et al. (1), *A. microcarpum* ethyl acetate fraction and methanolic fraction were tested in isolation or in combination with antibiotics (amikacin, gentamicin, ciprofloxacin, and imipenem) against *E. coli, P. aeruginosa*, and *S. aureus*. All extracts revealed low antibacterial activity against multiresistant strains (MIC = 512 μg/mL). However, the association of natural products with antibiotics presented a synergistic effect against the multiresistant *E. coli* strain. Moreover, the extract and ethyl acetate fraction, in conjunction with amikacin and gentamicin, also demonstrated synergism with imipenem against *S. aureus*.

Pereira et al. (203) evaluated three extracts (20 mg/mL) from *A. humile* leaves (ethanol, butanol, and hexane fractions) against *S. mutans, S. aureus*, and *A. actinomycetemcomitans* by agar diffusion and broth microdilution. Inhibition halos ranged from 9 to 19 mm, with the best result being obtained for ethanolic extract against *S. aureus*. MIC evaluation revealed a 0.50 mg/mL value for *S. mutans*, 1 mg/mL for *S. aureus*, and 3.50 mg/mL for *A. actinomycetemcomitans*. Chloramphenicol (10 μg/mL) presented halos ranging from 19 to 36 mm.

##### Antifungal Properties

The increase in fungal resistance and the incidence of infections has led to the realization of tests aiming to evaluate the antifungal potential of species from the *Anacardium* genus against primary and opportunistic pathogenic fungi.

*A. occidentale* stem bark extract (1:1 to 1:512 mg/mL) presented action against *Candida tropicalis* and *Candida stellatoidea* strains with inhibition halos ranging from 17 to 12 and 18 to 12 mm, respectively, where the chlorhexidine gluconate control obtained halos ranging from 12 to 22 mm (204). Bahadur et al. (205) found that *A. occidentale* cashew bark methanol extract (150, 200, and 300 ppm) reduced conidia germination (11% at 300 ppm) of *Erysiphe pisi* in humid chambers for analysis under the microscope. Kolaczkowski et al. (190) assessed the *A. occidentale* methanol extract (aerial parts) effect against *Candida glabrata*, by broth microdilution, obtaining a MIC of 0.08 mg/mL, while that of the control drug fluconazole was 0.008 mg/mL.

*A. occidentale* burnt cashew pulp extract was evaluated against fungi from the *Fusarium* genus. Disc diffusion assay revealed the action of burnt cashew pulp extract (5 mg/mL) on *Fusarium oxysporum* (±30%), *Fusarium moniliforme*, and *Fusarium lateritium* (±60%) growth decrease. KHCO_3_ (20 mg/mL) control had a zone ranging from ±15% to ±28%, and the Cercobin fungicide (10 ppm) had a zone ranging from ±25 to ±30% (206).

Harsini (207) observed through colony counting that rinse solutions made from the *A. occidentale* bark ethanol extract (1, 2, 3, 4, and 5%) influenced *C. albicans* adherence to acrylic resin. The number of colonies ranged from 1757.50 to 670.00 CFU/mL, showing better results than the control (1912.50 CFU/mL).

Santos et al. (159) observed that *A. occidentale* leaf and bark hydroalcoholic extracts (70%) had their action improved when exposed to gamma irradiation (0, 5.0, 7.5, and 10 kGy). Disc diffusion assays showed that, against *C. albicans*, the extracts (2,000 μg/disc) presented halos ranging from 14 to 0 mm and from 58 to 0 mm for bark and leaves, respectively. *A. occidentale* tincture (200 mg/mL) obtained from a Homeopathic Pharmacy was tested by microdilution and presented action against *C. albicans* and *Candida krusei* with a MIC value of 100 mg/mL. No growth was observed with the control nystatin (100,000 IU/mL) (187). *A. occidentale* dried leaf ethanolic extract (20 mg/mL) was tested by Anand et al. (80) against *C. albicans* presenting 15.67 mm inhibition halos, with gluconate (CHX) and povidine iodine (PI) presenting 14.67 and 19.67 mm, respectively. In the microdilution assay (10,000 μg/mL), the MIC and Minimum Fungicide Concentration (MFC) of the extract obtained a value of 1,250 μg/mL (CHX-−5 μg/mL and PI-−40 and 80 μg/mL, respectively). In the biofilm assay, the density for the extract obtained was of 0.107 nm, with a potent action compared to the controls (CHX: 0.102 nm and PI: 0.186 nm).

Muzaffar et al. (139) tested anacardic acid (0 to 100 μM) against *Magnaporthe oryzae*. A strong conidial germination inhibition was observed by counting colony forming units in samples treated with anacardic acid (75 μM-−70%), while no inhibition was observed in the control containing dimethyl sulfoxide (DMSO-−0.1%).

Mahata et al. (208) evaluated the cardanol activity extracted from *A. occidentale* cashew, and its derivatives against *C. albicans*. The best MIC values were obtained for 4-[(4-cardanyl)azo] benzoic acid (CABA) hydrogel derivatives (8 μg/mL), followed by Self-assembled CABA (16 ug/mL) and cardanol (64 μg/mL). Drastic damages (lysis) caused by products in the fungus's membrane and cell wall were observed using scanning electron microscopy, especially by the CABA compound.

*A. humile* dried leaf (50 and 400 μg/mL) hydroalcoholic extract and its fractions (hexane, dichloromethane, ethyl acetate, and isobutanol) presented activity against *C. albicans*. Microdilution test revealed strong inhibition at 400 μg/mL for both extracts and fractions (133).

Investigations reporting the antifungal activity of *Anacardium* species mostly highlighted the *A. occidentale* species, in a similar manner to the antibacterial activity. The hydroethanolic extract was the most commonly tested extract type with the bark being the most commonly investigated plant part through microdilution and against opportunistic *Candida* spp. pathogens. No *in vivo* studies have been reported and no reports have been found against dermatophyte fungi. As observed in ethnobiological research, the medical use of this genus is the treatment of gastrointestinal symptoms and skin disorders and, therefore, studies carried out involving fungi which act with this pathogenesis profile are of extreme relevance.

### Anticancer Activity

Cancer is the major cause of death worldwide, researchers are working to develop more therapeutic components for cancer treatment with less side effects. Plants are the main sources of pharmacologically active molecules, used for therapeutic purposes (209–211).

Taiwo et al. (26) conducted a study with Nigerian *A. occidentale* leaves in cultured HeLa cells. Four isolated compounds, zoapatanolide A, agathisflavone, anacardicin, and methyl gallate, were identified, and authors found that these components exhibited HeLa cell viability reduction in dose-dependent manner, although with distinct efficiencies: zoapatanolide A > anacardicin > agathisflavone > methyl gallate. The cytotoxic potential of zoapatanolide A is well-documented in literature (212). This class of compounds act as Michael acceptor for the cysteines thiol groups, covalently modifying proteins (213, 214). Kubo et al. (215) found that the biflavonoid, agathisflavone, has antiproliferative activity against Jurkat cells (IC_50_ = 4.45 μM), although other compounds isolated from *A. occidentale* juice have also revealed cytotoxic abilities, such as anacardicin, gallic acid, and other salicylic acid derivatives. The agathisflavone effect in several cancer cell lines (colon, lung, renal, breast, and ovarian cancer) was assessed, but only a marginal activity was stated, while to the methylated derivatives promissory effects were listed against these cancer cells (216) and even against chronic myeloid leukemia cell line K562 (217). Later, the agathisflavone effect on leukemia cells growth was further studied by Konan et al. (218); this compound induced lymphopenia *in vivo* and selectively triggered apoptosis. In addition, it was also stated that in Jurkat cells the antiproliferative ability of agathisflavone is more effective than on acute promyeloid leukemia (APL) cell line HL60, with IC_50_ of 2.4 and 11.03 μg/mL, respectively. On the other hand, the identification by liquid chromatography–mass spectrometry (LC-MS) of the cashew nut shell liquid purified from Indian *A. occidentale*, revealed a chemical composition of cardanol, anacardic acid, and methyl cardol. It inhibited HeLa cells proliferation, triggered moderate mitotic block and HeLa cells apoptosis, besides to accelerate wound closure in L929 cells, without causing toxic effects on normal cells (196).

Kishore et al. (219) reported that anacardic acid enhance aurora kinase A activity through induction of structural changes. This compound exerted cytotoxic effects on several human cancer cell lines *in vitro*. In view of histone acetyltransferase (HAT) inhibition, cashew nut shell liquid, anacardic acid, and their derivatives were assessed for tumor suppressing effects. There were no-mutagenic effects up to 0.003% with and without *S. typhimurium* strains metabolic activation (220). Anacardic acid also inhibited cardiomyocytes hypertrophy in isolated neonatal rat in response to phenylephrine or urocortin. Anacardic acid was also as effective as Spiruchostatin A (221).

Anacardic acid-induced Aurora kinase A autophosphorylation was shown in an *in silic*o approach, and this effects was attributed to its ability to bind and induce structural changes on the enzyme (219). Furthermore, Schultz et al. (222) stated that anacardic acid displayed effective inhibition toward estrogen receptor alpha (ERα)-expressing breast cancer cells proliferation, regardless of endocrine/tamoxifen sensitivity, while no effect was observed in ERα-negative cells. In addition, cell cycle progression inhibition and apoptosis induction in ERα -expressing cells was stated ERα-dependently. In short, as anacardic acid inhibited ERα-expressing breast cancer cells proliferation, but not the primary HuMECs, this finding reveals of utmost interest for further delineation of medical actions in cancer therapy.

Besides, marked effects were also reported by Sukumari-Ramesh et al. (223) on pituitary adenoma cells. Anacardic acid triggered polymerase cleavage induction, sub-G1 arrest, and annexin-V expression, reduced survivin and X-linked inhibitor of apoptosis protein and anti-apoptotic proteins expression, all associated with cell survival. However, carbobenzoxy-valyl-alanyl-aspartyl-(*O*-methyl)-fluoromethylketone failed to revert anacardic acid-induced cell death. Moreover, Chandregowda et al. (224), tested diverse benzamide derivatives synthesized for cytotoxic capacity on HeLa cells, being these compounds classified as potent as garcinol, with interesting IC_50_ values.

## Conclusions and Future Perspectives

*Anacardium* plants have extensively been largely reported for its antioxidant, anti-inflammatory, anticancer, and antimicrobial effects. A number of *in vitro* studies have been reported with promising results. On the other hand, the anticancer potential of *Anacardium* secondary metabolites is also quite prominent. Thus, *Anacardium* plants should be further studied to better elucidate their therapeutic potential not only in the *in vitro* and *in vivo* studies, but also the clinical application.

## Author Contributions

JS-R: conceptualization. JS-R, MM, AJ, WC, and NM: reviewed and editing. All authors: validation investigation, resources, data curation, writing, read and approved the final manuscript, and contributed equally to the manuscript.

## Conflict of Interest

BÖ was employed by the company Bioactive Research and Innovation Food Manufac. Indust. Trade Ltd. The remaining authors declare that the research was conducted in the absence of any commercial or financial relationships that could be construed as a potential conflict of interest.
